# Genome sequence of *Halovibrio* sp. HP20-59 as a promising polyhydroxybutyrate producer

**DOI:** 10.1007/s00253-025-13647-3

**Published:** 2026-01-10

**Authors:** Shivani Adhvaryu, Jana Kiskova, Maria Piknova, Veronika Farkasova, Iva Buchtikova, Xenie Kourilova, Martin Kizovsky, Marketa Benesova, Ota Samek, Stanislav Obruca, Peter Pristas

**Affiliations:** 1 Department of Microbiology, Institute of Biology and Ecology, Faculty of Science, Pavol Jozef Safarik University of Kosice, Srobarova 1014/2, 04180 Kosice, Slovakia; 2 Institute of Food Science and Biotechnology, Faculty of Chemistry, Brno University of Technology, Purkynova 464/118, 61200 Brno, Czech Republic; 3 Institute of Scientific Instrumentsof the , Czech Academy of Sciences, Brno, 61264 Czech Republic

**Keywords:** Halophiles, *Halovibrio*, *Oceanospirillales* order, Polyhydroxyalkanoates, PHA synthase (PhaC), Raman spectroscopy

## Abstract

**Abstract:**

Since plastics pose the greatest threat to humanity, it is essential to find an economic and sustainable solution to combat environmental pollution. In this study, the ability of polyhydroxyalkanoates (PHA) production by the halophilic bacterium *Halovirbrio* sp. HP20-59 in the presence of different carbon sources was examined. The strain showed a selective substrate preference, with the highest PHA production (reaching up to 73% of cell dry weight) in the presence of galactose, while fructose, arabinose, glycerol and xylose resulted in lower accumulation. Phylogenetic analysis based on the 16S rRNA gene sequence and whole-genome sequencing confirmed the HP20-59 strain as a novel species within the *Oceanospirillales* order. Draft genome showed a size of 4,165,370 bp with a GC content of 55.1% and a complete set of *pha* genes. The comparative analysis of the *phaC* gene identified a 638 amino acid-long class I poly(R)-hydroxyalkanoic acid synthase, showing 91% similarity to *Halovibrio variabilis* and 89% similarity to species within the *Vreelandella* genus, suggesting a possible horizontal gene transfer of the *pha* gene cluster. These findings highlight the unique genetic and metabolic characteristics of *Halovibrio* sp. HP20-59, making it a promising candidate for industrial PHA production and a valuable resource for research on sustainable biopolymers.

**Key points:**

- *The first study of PHB production by the halophilic Halovibrio spp.*
- *The highest level of PHB production observed using glucose, galactose, and sucrose.*
- *phaCAB operon possibly acquired by horizontal gene transfer from Vreelandella sp.*

**Supplementary Information:**

The online version contains supplementary material available at 10.1007/s00253-025-13647-3.

## Introduction

The industrial economic boom started after the discovery of polyethylene and forced everyone to use petrochemical plastics from everyday kitchen appliances to electronics, packaging and medicinal instruments. Human relentless demand and consumption of plastic have exceeded limits, resulting in plastics being the major contributor to recent environmental changes and disruption of the Earth’s ecosystem (Ali et al. 2022). Abdallah et al. (2025) stated in their review that global plastic production reached 400.3 million tons in 2022, primarily in the form of polyvinyl chloride (PVC) and polypropylene (PP). The growing accumulation of plastic waste has severe environmental impacts, as degradation leads to microplastics (< 5 mm) and nanoplastics (< 1 μm), posing a significant threat to marine life (Chouchene et al. 2023). Despite advances in recycling, landfill, and incineration, these plastic waste management methods do not fully resolve sustainability challenges related to waste accumulation (Abdallah et al. 2025).

The plastic solution relies on exploring a bio-based economy having the potential to produce bio-based plastics and to shift the focus from fossil fuels to other renewable resources such as sugars from sugarcane, corn, cellulose, starch, lignin, fats, oils, organic waste, methane, and carbon dioxide (Guleria et al. 2022; Mukherjee and Koller 2023). In addition, some microorganisms are also able to produce biodegradable, biocompatible, and structurally diverse biopolymers, such as polyhydroxyalkanoates (PHA), compared to conventional fossil fuel-based plastics (Zhou et al. 2023).

PHA are produced by microorganisms as a storage material in the absence of nutrients such as nitrogen, sulfur or phosphorus and in the presence of excess carbon sources (Rekhi et al. 2022). Chemically, PHA are polyesters of hydroxyalkanoic acids linked by an ester bond of varying length with a molecular weight from 50 to 1000 kDa (Reddy et al. 2003). Bacterial cells store PHA in the form of round granules of different sizes. These granules are insoluble and osmotically inactive, with their quantity and size influenced by the bacterial strain and growth conditions (Shen et al. 2019; Zhou et al. 2023). PHA production in microbes proceeds through three important pathways, in which amino acids, sugars and fatty acids were converted to the final product PHAs employing acetyl-CoA or acyl-CoA as the intermediate species. This process involves a series of enzymatic reactions. Commonly, PhaA (3-ketothiolase), PhaB (acetoacetyl Co-A reductase) and PhaC (PHA synthase) are the main biosynthetic enzymes, and PhaZ (PHA depolymerase) is involved in either intracellular or extracellular PHA degradation (Saravanan et al. 2022). PHA synthase (coded by the *phaC* gene) is considered a key enzyme in the biosynthesis process and plays an important role in the polymerization of (R)−3-hydroxyalkanoyl coenzyme A to PHAs. The classification of PHA synthase involves four classes, where class I and II consist of a single PhaC subunit and class III and IV consist of two different types of the main PhaC subunit and one additional subunit, PhaE (class III) or PhaR (class IV) (Zher Neoh et al. 2022). Poly(R)−3-hydroxybutyric acid (PHB) is the most prominent member of the PHA family (Najar et al. 2024).

Bioplastics of the PHA family are obtained by microbial consumption of various carbon resources and have the properties of an environmentally friendly, biodegradable and renewable material. In order to reduce costs and achieve sustainable goals, agricultural waste products and wastewater can be used as a carbon source for PHA production (Adeleye et al. 2020).

The incredible market potential and commercial applications of PHAs encounter the problem of high costs and recently halophiles have received significant scientific interest as a promising and cost-effective alternative (Mitra et al. 2020). Halophiles can be unique candidates for PHA production due to the following reasons:

(i) Their high salinity requirement reduces the possibility of microbial contamination in the fermentation process
(ii) PHA recovery cost is reduced as hypotonic cell lysis releasing PHA granules induced by ordinary water can be used instead of expensive chemicals
(iii) Substrate cost can be reduced as halophiles can utilize inexpensive raw materials (Mitra et al. 2020; Angra et al. 2023)

 Studies have reported that halophiles produce a range of metabolites with industrial applications, including enzymes, compatible solutes, exopolysaccharides, β-carotene, or biofuels which can be considered interesting co-products to PHA further improving the economic aspects of the biotechnological process (Oren 2010).

The aim of this study was to investigate PHA production by the halophilic strain HP20-59, a novel and promising bacterium isolated from brine at the former salt mine Solivar near Prešov (Slovakia). This strain was taxonomically classified as a member of the genus *Halovibrio*. Given its remarkable potential for PHA biosynthesis, we further examined the influence of various carbon sources on PHA accumulation except glucose (subjected to another study) and sought to uncover the molecular mechanisms underlying PHA production in this promising microorganism.

## Materials and methods

### Sampling and cultivation analysis

The flowing brine sample from the borehole was collected in a 50 mL sterile tube from the former salt mine Solivar near Prešov city, Slovakia (48°59′0.706″ N, 21°16′59.124″ E) in September 2020. The pH, temperature, total dissolved solids (TDS) and conductivity of the brine were determined according to our previous studies (Nosalova et al. 2022; Brestovicova et al. 2024) and immediately transported back to the laboratory for further analysis.

One hundred microliters of water sample was immediately inoculated onto R2A medium (Sigma-Aldrich, USA) supplemented with 5% NaCl *(w/v)* (CentralChem, Slovakia) and cultivated at laboratory temperature (23–25 °C) for 48–72 h until the bacterial colonies were observed and colony-forming units (CFUs) per mL were calculated. Individual colonies were re-streaked on the same medium to obtain pure culture and subjected to further analysis.

Firstly, all obtained isolates were de-replicated using Matrix-Assisted Laser Desorption/Ionization Time-Of-Flight Mass Spectrometry (MALDI-TOF MS) according to the methodology described in our previous study (Nosalova et al. 2022; Kisková et al. 2023). At least one representative from each MALDI group was selected and subjected to the 16S rRNA gene sequence analysis. Subsequently, the HP20-59 isolate was selected and subjected to PHA production and whole-genome sequence analysis.

#### Colony characteristics, cell morphology, and growth of the HP20-59 isolate

The characteristics of bacterial colonies cultivated on the R2A medium were observed using the Leica EZ4 D stereo microscope (Leica Microsystems, Germany). Bacterial cells were stained by Gram’s method and observed using the optical microscope Motic BA (MOTIC Scientific, Hong Kong). Cellular morphology was further examined using transmission electron microscopy. The overnight bacterial culture (OD600 0.6–0.8) in R2A broth (HiMedia Laboratories Pvt.Ltd., India) was adhered to formvar-coated slot copper grids. The sample was washed with distilled water, negatively stained using 2% uranyl acetate (w/v), washed again, and air dried. The cellular morphology was observed using a JEM 1230 transmission electron microscope (JEOL, Japan) at an accelerating voltage of 80 kV.

To determine the optimal salinity for bacterial growth, the HP20-59 isolate was cultivated in R2A broth with NaCl concentrations of 0, 2.5%, 5%, 10%, 15%, 20%, 25%, and 30% at a temperature of 25 °C with constant shaking at 160 rpm. Absorbance measurements were performed using a UV-6300PC spectrophotometer (VWR, USA) every 12 h for 3 days until the bacterial culture reached a stationary phase.

#### Identification based on the 16S rRNA gene sequence analysis

The HP20-59 strain was overnight cultivated at 25 °C in R2A broth supplemented with 5% NaCl. Total DNA was extracted using the NucleoSpin Microbial DNA Kit (Macherey–Nagel, Germany) according to the manufacturer’s instructions. The quality of the obtained DNA was verified using electrophoresis in a 1% agarose gel stained with ethidium bromide (0.1 µg L^−1^) and examined using the ChemiDoc™ XRS + System (BIO-RAD, USA). The DNA concentration and purity were assessed spectrophotometrically using the NanoDrop 2000c Spectrophotometer (Thermo Scientific, USA).

The 16S rRNA gene was amplified using the TaqCore kit/high yield (Jena Bioscience, Germany). The 50 μL of PCR mixture containing 1x Crystal Buffer, 200 μM dNTP, 200 nM of each universal primer (fD1 5′-AGAGTTTGATCCTGGCTCAG-3′, rP2 5′-ACGGCTACCTTGTTACGACTT-3′) (Weisburg et al. 1991), 0.025 Unit μL^−1^ Taq DNA polymerase, and 1 µL of 50 ng DNA template was prepared. The PCR amplification was performed in Mastercycler® pro S (Eppendorf, Germany) under the following conditions: initial denaturation at 95 °C for 5 min, then 35 cycles of denaturation at 95 °C for 1 min, annealing at 56 °C for 1 min and extension at 72 °C for 1.5 min, and final extension step at 72 °C for 10 min. Quality and quantity of PCR amplicons were checked using 1% agarose gel electrophoresis stained with ethidium bromide (0.1 μg L^−1^) in 1 × TAE buffer through ChemiDocTM XRS + System. DNA concentration and purity were checked using NanoDrop 2000c Spectrophotometer. PCR amplicons were purified by ethanol precipitation and sequenced by the Sanger method with the SEQme s.r.o. sequencing service (Dobříš, Czech Republic).

Raw sequences were processed using the BioEdit v.7.2.5 software (Hall 1999) and compared against the NCBI Genebank rRNA/ITS database using the BlastN tool (Donkor et al. 2014). The obtained 16S rRNA gene sequence was deposited into GenBank under accession number PP717853.

The MEGA v11.0.13 software was used for the phylogenetic analysis (Tamura et al. 2021). The HP20-59 16S rRNA sequence was aligned with closely related 16S rRNA gene sequences obtained from the GenBank database using the ClustalW tool. A phylogenetic tree was constructed using the Maximum Likelihood method with 500 bootstrap replications and evolutionary distances were calculated using the Kimura 2-parameter model (Kimura 1980).

#### Rapid detection of PHA production by Nile Blue staining

A preliminary assessment of the PHA accumulation ability of the HP20-59 isolate was performed via Nile Blue A (NB) staining. The isolate was inoculated onto a mineral salt solution (MSS) containing 5% NaCl, 0.7% MgCl_2_·6H_2_O, 0.96% MgSO_4_·7H_2_O, 0.036% CaCl_2_, 0.2% KCl, and 0.006% NaHCO_3_ (CentralChem, Slovakia) supplemented with 2% glucose, 0.01% yeast extract, 0.01% peptone, 1.5% agar, and 1 µg mL^−1^ NB, and incubated at a temperature range of 20–25 °C for 72 h. After incubation, fluorescence was observed under UV light using a UV Transilluminator (Cleaver Scientific, UK).

#### PHA accumulation examination using Raman spectroscopy

To examine the PHA production, dried cells of the HP20-59 strain were analyzed using a Renishaw inVia Raman Spectrometer (Renishaw plc., UK) with a 785 nm single-mode diode laser as the excitation source. The cells were analyzed using a 50 × objective lens, focusing the laser beam into a spot measuring 2 µm × 10 µm. To capture the key spectral features of PHA, the measurement was centered at 1300 cm^−1^, ensuring the inclusion of its three dominant peaks. Five spectra were obtained for each sample, with an integration time of 1 s and 10 accumulations. The obtained spectra were processed using the Savitzky–Golay algorithm (order 1, frame length 7) for smoothing, rolling circle filtering (radius 300 cm^−1^, passes 30) for fluorescent background correction, and then normalized at 1002 cm^−1^ (phenylalanine).

#### The effect of various carbon sources on PHA production by the HP20-59 isolate

A two-step cultivation method was used to assess the effect of carbon sources on PHA production. The HP20-59 strain was initially cultivated in 50 mL of mineral salt solution (MSS) enriched with 0.5% yeast extract (Sigma-Aldrich, Germany) and 0.5% peptone (Sigma-Aldrich, USA) in a 100 mL Erlenmeyer flask. The pH of the medium was adjusted to 7, and the culture was incubated at 30 °C with shaking at 200 rpm for 24 h.

After the initial cultivation, 10% (5 mL) of the inoculum was transferred into a 250 mL Erlenmeyer flask containing 100 mL of production medium based on MSS, supplemented with 0.1% yeast extract and 0.1% peptone. As in the previous step, the pH was adjusted to 7 to maintain optimal growth conditions. Following sterilization, fructose, xylose, maltose, sucrose, ribose, galactose, arabinose, mannitol, lactose, cellobiose, mannose, or glycerol was added at a final concentration of 20 g L^−1^ using a 0.20 µm non-pyrogenic sterile microfilter (Sarstedt, Germany). The culture was then incubated in a 250 mL Erlenmeyer flask at 30 °C with shaking at 200 rpm for 72 h to support microbial growth and PHA production.

After incubation, the bacterial culture was centrifuged at 8000 × g for 5 min. The pellet was dried at 55 °C until a constant weight was achieved, after which it was weighed and prepared for PHA analysis using Raman spectroscopy and gas chromatography.

#### PHA analysis using gas chromatography-flame ionization detector (GC-FID)

The amount of PHA in the dried cells and the produced monomer composition were identified as methyl esters of particular 3-hydroxyacids by gas chromatography with a flame ionization detector (GC-FID) according to Obruca et al. (2014). Nearly 8 to 11 mg of dried biomass was suspended in 1 mL of chloroform and 0.8 mL of esterification mixture in a small crimp neck 2 mL vial. The esterification mixture, acting as an internal standard, contained 15% H_2_SO_4_ dissolved in methanol along with 5 mg mL^−1^ benzoic acid (internal standard). The vials were tightly sealed and incubated in a dry bath at 94 °C for 3 h to facilitate methanolysis, during which intracellular PHA was converted into hydroxy-carboxylic acid methyl esters. After the reaction, a 4 mL screw-cap vial with 0.5 mL of 50 mM NaOH was prepared. The entire contents of the small crimp vial were then poured into it and shaken vigorously to ensure thorough mixing. In this way, a phase separation was established and the lower organic phase of 50 µL was transferred into a new 2 mL small screw cap vial containing 0.9 mL of isopropyl alcohol and used in GC measurement. The Trace GC Ultra instrument (Thermo Fisher Scientific, USA) was used to measure the amount of fatty acid methyl esters. The flame ionization detector has the set-up of a Stabilwax (Restek) column (Crossbond Carbowax polyethylene glycol) (30 m × 0.32 mmlD × 0.5 µm df, max. prog. temp. 260 °C, min bleed at 250 °C) for the measurement. Commercially available polymers poly(3-hydroxybutyrate) (PHB) and poly(3-hydroxybutyrate-co-3-hydroxyvalerate) (PHBHV) with 12 mol% of 3-hydroxyvalerate (Merck, Germany).

#### The whole-genome sequence analysis

To gain a deeper understanding of the genomic features of the HP20-59 strain, whole genome sequencing was performed using the Illumina NovaSeq 6000 platform by paired-end approach (2 × 150 bp) with the S4 PE150 XP kit (Eurofins Genomics Europe Sequencing GmbH, Konstanz, Germany). The raw reads were processed using tools implemented in the Unipro UGENE v47.0 bioinformatics software (Okonechnikov et al. 2012; Golosova et al. 2014). The sequence quality was assessed using the FastQC v0.11.9 tool, and the Trimmomatic v0.39 tool was used to trim sequences with an average quality score below 20. High-quality paired-end reads were assembled de novo using the SPAdes v3.12.0 tool. Finally, the contigs shorter than 200 bp were removed from the draft genome. The draft genome sequences were deposited in the GenBank database under accession number NZ_JAWJUW000000000.2 (BioProject: PRJNA224116, BioSample: SAMN37745899).

The genomic relatedness of the HP20-59 strain to representatives of the *Oceanospirillales* and *Desulfovibrionales* orders was determined using the in silico digital DNA:DNA hybridization (dDDH) analysis performed by Type (Strain) Genome Server (TYGS) with the species cut-off value of 70% (Meier-Kolthoff and Göker 2019) and by Average Nucleotide Identity (ANI) and Average Amino Acid Identity (AAI) values of 95% species delineation calculated using the JSpeciesWS server (Richter et al. 2016) and Kostas Lab AAI calculator (Goris et al. 2007), respectively. The phylogenetic position of the HP20-59 strain was evaluated using the Genome BLAST Distance Phylogeny (GBDP) tree constructed using TYGS.

The HP20-59 genome was annotated by Rapid Annotation using Subsystem Technology (RAST) (Brettin et al. 2015) and genes involved in PHA production were identified. Contigs carrying *pha* genes were analyzed using the Proksee server (Grant et al. 2023), with Alien Hunter and mobileOG-db tools to identify horizontal gene transfer (HGT) regions. The RAST results were used to study the genomic background of the *pha*C gene. The evolutionary relatedness of the PHA synthase was assessed within closely related sequences using the phylogenetic tree constructed by the MEGA v11.0.13 software with the Neighbor-Joining method, the Kimura 2-parameter model, and 1000 bootstrap replications.

#### Comparative analysis of PHA production efficiency and PHA-related gene clusters

A comparative analysis based on data available from scientific studies was performed to evaluate the efficiency of PHA production in different bacterial strains under varying cultivation conditions. Specifically, parameters such as the type of carbon source supplied and the cultivation method employed were considered.

Based on PHA production data available in scientific studies, five genomes of different bacteria deposited in GenBank were selected and the gene structure was determined based on the available GenBank annotation.

## Results

The sampling site, Solivar near Prešov, is located in eastern Slovakia in a former salt mine (Kovac et al. 2017). During the September 2020 sampling in Prešov, Slovakia, the brine temperature was 12.3 °C, with a pH of 6.5 and 311 g L^−1^ of total dissolved solid content. Approximately 200 CFU ml^−1^ of cultivable bacteria were detected, including multiple true halophilic isolates, which were tested for PHA production (Nosalova et al. 2022; Brestovicova et al. 2024). The HP20-59 isolate was selected for further experiments.

### Colony characteristics, cell morphology, and growth of the HP20-59 isolate

**Fig. 1:**
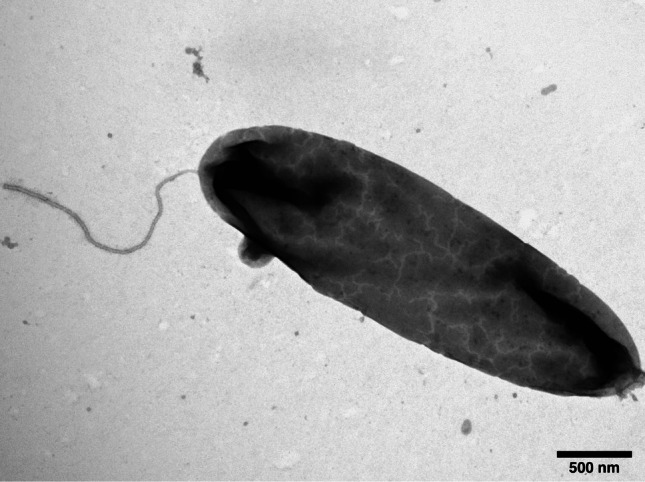
*Halovibrio* sp. HP20-59 under transmission electron microscopy

The HP20-59 isolate is a Gram-negative bacterium with a rod-shaped morphology (Fig. S1A). The bacterium forms white, round colonies with smooth edges (Fig. S1B). Cells are characterized by the presence of a single polar flagellum (Fig. 1).

**Fig. 2:**
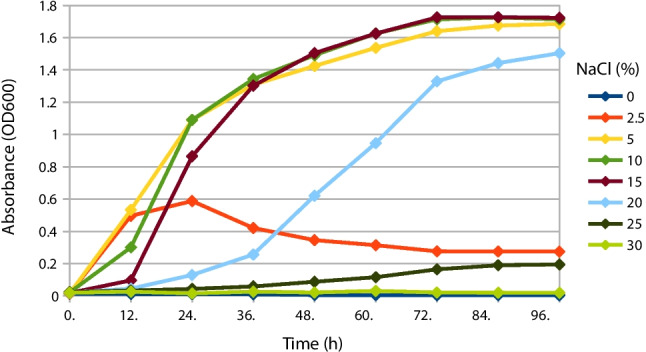
The growth of the HP20-59 isolate observed in presence of different salt concentration

The isolate grew best at 5% NaCl in the culture medium. Salt concentrations of 10% and 15% NaCl also provided suitable growth conditions. Very high concentrations of NaCl (25% and 30%) significantly inhibited its growth. The isolate showed no growth at 0% NaCl, indicating that the HP20-59 isolate is a halophilic (not a halotolerant) bacterium (Fig. 2).

### PHA screening using Nile Blue A staining

The Nile blue A staining was used to detect the PHA production by the HP20-59 isolate (Fig. S2). The fluorescence intensity was positively correlated with the amount of PHA synthesized. Based on these findings, the HP20-59 isolate was selected for detailed analysis using Raman spectrometry and GC-FID.

### The identification and phylogenetic relatedness of the HP20-59 isolate based on the 16S rRNA gene sequence

**Fig. 3:**
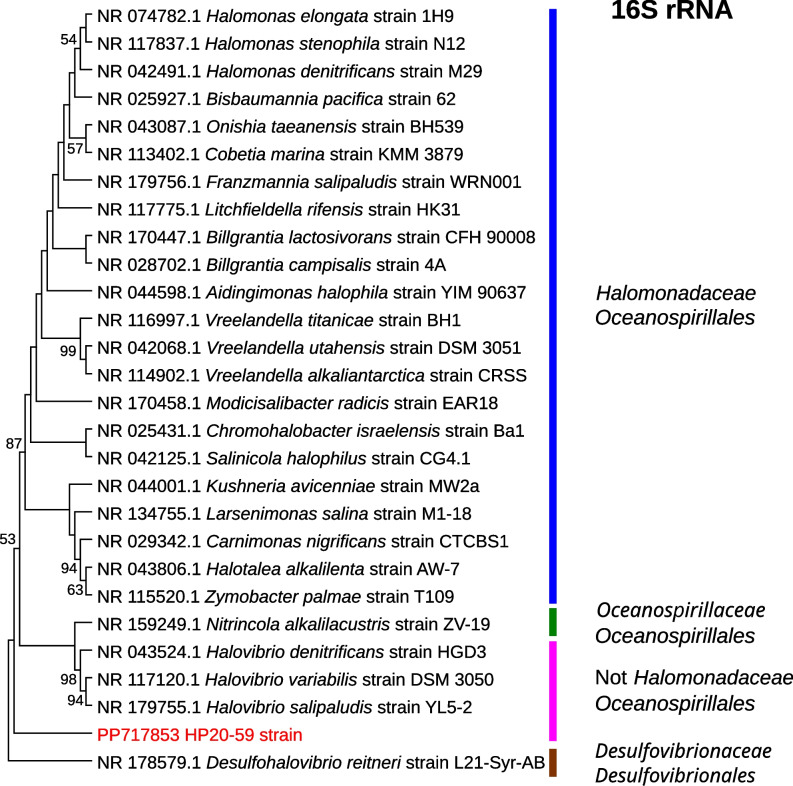
The phylogenetic placement of the HP20-59 isolate within the *Oceanospirillales* order based on the 16S rRNA gene sequence comparison. The phylogenetic tree was constructed using the maximum likelihood method and the Kimura 2-parameter model. Bootstrap values (≥50%) calculated based on 500 replications are shown at branch nodes. The *Desulfohalovibrio reitneri* strain L21-Syr-AB 16S rRNA sequence was used as an outgroup. The HP20-59 strain is highlighted in red color

The 16S rRNA gene sequence of the HP20-59 strain showed the highest similarity (98.95%) to *Vreelandella utahensis* DSM 3051 (NR_042068.1) and *Vreelandella neptunia* (NR_114902.1) CRSS (98.82%) when compared against the GenBank 16S rRNA gene sequence database. Identity values are too low to reliably assign the HP20-59 isolate to any bacterial species. The phylogenetic analysis using type strain sequences of several species belonging to the *Halomonadaceae* family and *Oceanospirillales* order placed the HP20-59 isolate in a separate branch, even outside the *Halomonadaceae* family and the *Halovibrio* genus belonging to the *Oceanospirillales* order (Fig. 3).

### Effect of different carbon sources on PHA production detected using Raman spectroscopy analysis

**Fig. 4:**
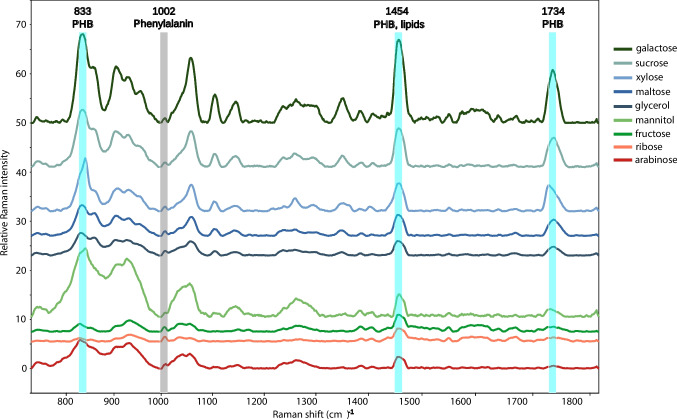
The comparison of Raman spectra of the HP20-59 isolate cultivated on different carbon sources. All spectra were processed, averaged within the groups, and stacked for better clarity. Typical peaks used for PHA quantification are marked

The PHA production by the HP20-59 strain was examined using 9 different carbon sources and PHA content was analyzed by Raman spectroscopy (Fig. 4). All samples appeared to contain detectable amounts of PHB, most notably the sample in which galactose was used as the carbon source. The Raman peaks of PHB were observed at 833 cm^−1^, 1454 cm^−1^, and 1734 cm^−1^, and the peak at 1734 cm^−1^ is considered the most accurate, as there was no interference with the vibrations of other cellular components, which demonstrated that the PHB was in an amorphous state. Cells cultivated with mannitol and xylose exhibited a PHB peak noticeably sharper and seemingly tilted to the left side (near the range of 1725 cm^−1^) (Fig S3). This finding suggests an increased level of PHB crystallization. Fig. S3 shows the relative Raman intensity of the PHB peak between 1730 and 1740 cm^−1^ depending on the different sugars used in the cultivation medium. The data obtained indicate that galactose is the best carbon source for PHA production by the HP20-59 isolate.

### The quantification and characterization of PHA produced by the HP20-59 isolate

The quantification and characterization of PHA produced by the HP20-59 isolate on various substrates were performed using GC-FID, and the results are summarized in Table 1. In all samples, only the peak corresponding to 3-hydroxybutyrate was detected (Fig. S4), indicating that under the tested cultivation conditions (i.e., without supplementation of alternative monomer precursors), this strain accumulated exclusively the homopolymer poly(3-hydroxybutyrate) (PHB). The highest PHA concentration (2.11 g L⁻^1^) was obtained when galactose was used as the sole carbon source, representing 73.76% of the total cell dry mass (2.865 g L⁻^1^) (Table 1). Moderate PHA titers in the range of 0.75–1.5 g L⁻^1^ were observed with mannitol, sucrose, xylose, and maltose, corresponding to PHA content in dry cell biomass of 37.08–52.25. In contrast, carbon sources such as fructose, ribose, arabinose, and glycerol supported only low or even undetectable PHA accumulation, which correlated well with Raman spectrometry data.

**Table 1:** The GC-FID data on PHA production in the HP20-59 isolate during cultivation using various carbon sources. The PHA content is expressed as an arithmetic mean value ± standard deviation (SD) of two measurements

**C-source ** **(20 g L**^**−**1^**)**	**Type of PHA**	**CDW (g L**^**−1**^) Mean ± SD	**PHA content in CDW (%)** Mean ± SD	**PHA titer (g L**^**−1**^**)** Mean ± SD
Galactose	PHB	2.87 ± 0.29	73.76 ± 2.84	2.11 ± 0.08
Sucrose	PHB	2.81 ± 0.11	52.25 ± 2.03	1.47 ± 0.06
Mannitol	PHB	2.85 ± 0.52	37.08 ± 14.11	1.05 ± 0.40
Maltose	PHB	2.48 ± 0.11	40.63 ± 1.34	1.01 ± 0.03
Xylose	PHB	1.71 ± 0.09	44.35 ± 12.43	0.76 ± 0.21
Glycerol	PHB	1.83 ± 0.04	27.63 ± 0.08	0.51 ± 0.00
Arabinose	PHB	1.42 ± 0.18	24.25 ± 5.13	0.34 ± 0.07
Fructose	PHB	2.00 ± 0.00	4.30 ± 0.02	0.09 ± 0.00
Ribose	PHB	0.52 ± 0.04	ND	ND

CWD cell dry weight, ND not detected

### Genome features of the HP20-59 strain

A total of 6,754,860 raw reads were obtained by whole-genome sequencing. The draft genome reached a size of 4,165,370 bp and it was assembled into 208 contigs containing 55.1% GC. The N50 was 238,926 bp while the L50 was 6 (Table S1). The genome contained 4091 coding sequences with 67 RNAs. Two plasmids were identified in the HP20-59 genome (NODE_7 with a size of 194,106 bp and NODE_36 with a size of 3246 bp).

According to the RAST subsystem annotation, amino acids and derivatives had the highest number of genes followed by protein metabolism, carbohydrates, cofactors, vitamins, prosthetic groups, pigments and respiration (Fig. S5). The RAST annotation identified a total of 19 osmotic stress response genes such as, choline and betaine uptake and betaine biosynthesis gene cascades and osmoprotectant ABC transporter YehZYXW.

### The phylogenomic placement of the HP20-59 isolate

**Fig. 5:**
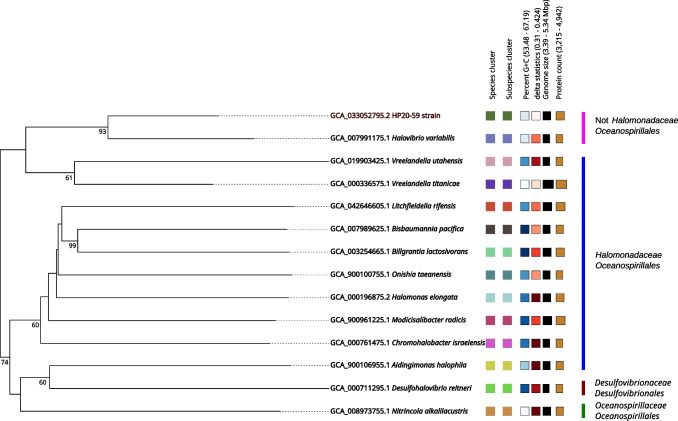
The phylogenetic placement of the HP20-59 strain within the *Oceanospirillales* order based on the Genome BLAST Distance Phylogeny (GBDP) analysis available at the TYGS. The numbers under branches are GBDP pseudo-bootstrap support values based on 100 replications. Leaf labels with different colors indicate species and subspecies clusters; the color range increases (from light to dark) based on genomic GC content and delta statistics. Different label sizes correspond to genome size and the number of proteins

The phylogenetic analysis based on whole genome sequencing placed the HP20-59 strain in a well-supported separate branch with *Halovibrio variabilis* NBRC 102410 (NZ_BJXV00000000.1, GCA_007991175.1) as a sister taxon within the GBDP tree; however, it is outside the *Halomonadaceae* family of the *Oceanospirillales* order (Fig. 5). The observed dDDH value (35%) and ANI and AAI values (< 95%) clearly indicate that the HP20-59 isolate represents a new species of the *Oceanospirillales* order (Table 2).

**Table 2:** dDDH values between genome of *Halovibrio* sp. HP20-59 and representatives of the *Oceanospirillales* and *Desulfovibrionales* order and pairwise comparison of the genome similarities based on ANI and AAI values

**Bacterial species**	***Halovibrio* sp. HP20-59**		
	**dDDH (d4, %)**	**ANI (%)**	**AAI (%)**
*Halovibrio variabilis* GCA_007991175.1	35.0	87.86	91.57
*Vreelandella titanicae* GCA_000336575.1	27.2	83.25	85.86
*Vreelandella utahensis* GCA_019903425.1*_*	21.7	66.03	50.96
*Halomonas elongata* GCA_000196875.2	19.7	71.12	69.48
*Billgrantia lactosivorans* GCA_003254665.1	19.7	71.60	69.94
*Bisbaumannia pacifica* GCA_007989625.*1*	20.2	71.41	68.59
*Litchfieldella rifensis* GCA_042646605.1	19.5	70.75	68.24
*Onishia taeanensis* GCA_900100755.1	19.6	70.96	67.90
*Chromohalobacter israelensis* GCA_000761475.1	20.3	70.19	66.17
*Modicisalibacter radices* GCA_900961225.1	21.0	70.38	66.04
*Aidingimonas halophila* GCA_900106955.1	18.9	69.52	67.37
*Nitrincola alkalilacustris* GCA_008973755.1	20.0	66.36	52.58
*Desulfohalovibrio reitneri* GCA_000711295.1	19.1	63.07	38.77

### Phylogenetic relationship of the *phaC*gene from* Halovibrio* sp. HP20-59

**Fig. 6:**
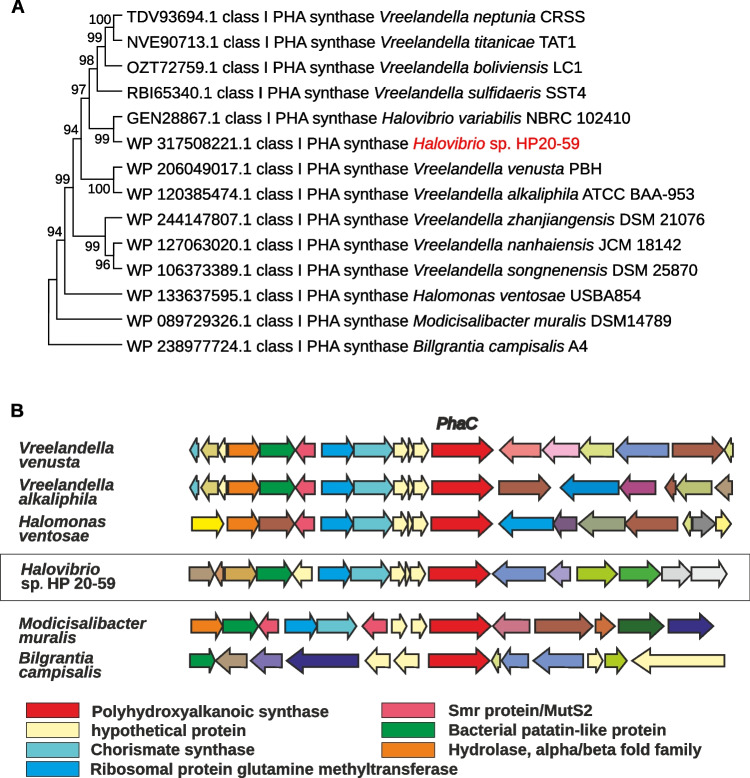
**A** The phylogenetic tree showing the relatedness of the HP20-59 class I PHA synthase to the *Vreelandella* spp. class I PHA synthases. The phylogenetic tree was constructed using the Neighbor-Joining algorithm, the Kimura 2-parameter model, and 1000 bootstrap replicons. **B** The comparison of genetic organization of the *phaC* gene context in the HP20-59 isolate and other members of the *Halomonadaceae* family

In the HP20-59 genome, a complete set of genes responsible for PHA production and utilization was identified (Table 3). *PhaA* and *phaB* genes were part of regions identified as HGT, and a part of the *phaC* gene was also included in the HGT region (Fig. S6) with slightly higher GC content (56.65%, 55.56%, and 56.32%) compared to the rest of the *Halovibrio* sp. HP20-59 genome (55.1%). The *phaC* gene encodes class I poly(R)-hydroxyalkanoic acid synthase, which was compared against related *phaC* sequences in the GenBank database using the blastp algorithm. The PhaC protein is 638 aa long and showed the highest similarity (91%) to the class I PHA synthase of *Halovibrio variabilis* (WP_146875779.1). The phylogenetic analysis clearly placed the class I PHA synthase of the HP20-59 strain within the *Vreelandella* species cluster (Fig. 6A). The close relationship between PHA synthase sequences supports the theory about HGT among these species, which is also confirmed by the very similar gene arrangement of *Halovibrio* sp. HP20-59 compared to *Vreelandella* spp., which differs from other *Halomonadaceae* species (Fig. 6B).

**Table 3:** Genes involved in the PHA synthesis pathway identified in the *Halovibrio* sp. HP20-59 genome

Accession No	Type	bp	Gene	EC No	Product
WP_317508792.1	CDS	1179	*phaA*	2.3.1.9	Acetyl-CoA C-acetyltransferase
WP_317508129.1	CDS	1188	*fadA*	2.3.1.16	Acetyl-CoA C-acyltransferase/3-ketoacyl-CoA thiolase
WP_317508680.1	CDS	1179	*fadA*	2.3.1.16	Acetyl-CoA C-acyltransferase/3-ketoacyl-CoA thiolase
WP_317507505.1	CDS	747	*phaB*	1.1.1.36	Acetoacetyl-CoA reductase
WP_317508221.1	CDS	1917	*phaC*	2.3.1.-	Class I poly(R)-hydroxyalkanoic acid synthase
WP_317507089.1	CDS	1260	*phaZ*	3.1.1.75	PHB depolymerase

### Comparative analysis of PHA production efficiency and PHA-related gene clusters

The comparative analysis showed that carbon source significantly affects the type and amount of PHA produced (Table 4). In addition, the cultivation method (e.g., shake flask vs fermentor) also appears to play a significant role in determining PHA yield. Genome annotation of well-studied PHA-producing isolates revealed the presence of genes related to PHA biosynthesis (*phaA*/*bktB*, *phaB*, and *phaC*) as well as PHA regulatory and degradation (*fadA* and *phaZ*) in all analyzed genomes (Table S2). Class I PHA synthase was identified in all genomes. Interestingly, multiple copies of PHA biosynthesis and degradation genes did not affect PHA yield such as in *Paraburkholderia sacchari* DSM 17165.

**Table 4:** PHA production by different bacterial species under various cultivation conditions

Microbial strain	Carbon source	PHA type	PHA content(wt%/g L^−1^)	Device	Reference
*Vreelandella boliviensis *LC21	Maltose	PHB	58.8/0.55	Shake flasks	Quillaguaman et al. 2005
*V. boliviensis *LC21	Butyric acid and sodium acetate	PHB	54/ND	Shake flasks	Quillaguamán et al. 2006
*V. boliviensis *LC21	Butyric acid and sodium acetate	PHB	88/ND	2-L fermenter	Quillaguamán et al. 2006
*V. boliviensis *LC21	Glucose or sucrose	PHB	~ 55/ND	2-L fermenter	Quillaguamán et al. 2006
*V. boliviensis *LC1	Volatile fatty acid	P(3HB-*co*−8.5 mol% 3HV)	70/16	2-L bioreactor	García-Torreiroa et al. 2016
*Halomonas. hydrothermalis*	*Jatropha* biodiesel byproduct	PHB	74.1/0.40	Shake flasks	Bera et al. 2015
*H. hydrothermalis*	*Jatropha* biodiesel byproduct and 0.35% SDCLA	P(3HB-*co*−81 mol% 3HV)	73.3/1.07	Shake flasks	Bera et al. 2015
*H. hydrothermalis*	glycerol	PHB	ND/2.61	Shake flasks	Dubey and Mishra 2022
*Halomonas shrimpha *IBTH01	Glucose	PHB	52/4.54	Shake flasks	Kong et al. 2024
*Marinobacter haeunpha *IBTM02	Glucose	PHB	55/4.25	Shake flasks	Kong et al. 2024
*Halomonas. halophila*	Glucose	PHB	81.5/4.58	Shake flasks	Kucera et al. 2018
*H. halophila*	Sucrose	PHB	75.4/4.85	Shake flasks	Kucera et al. 2018
*H. halophila*	Cellobiose	PHB	90.8/2.59	Shake flasks	Kucera et al. 2018
*H. halophila*	Spent coffee grounds	PHB	61.95/2.17	Shake flasks	Kucera et al. 2018
*H. halophila*	Molasses	PHB	64.06/2.57	Shake flasks	Kucera et al. 2018
*H. halophila*	Cheese whey hydrolysate	PHB	38.32/3.26	Shake flasks	Kucera et al. 2018
*H. marina*	Glucose	PHB	> 59/ND	Shake-flasks	Biswas et al. 2009
*H. marina*	Glucose and 0.1% (w/v) valerate	P(3HB-*co*−12.8 mol% 3HV)	80/ND	Shake-flasks	Biswas et al. 2009
*Cobetia amphilecti *MC34	Glucose	PHB	35/0.6	Shake flasks	Christensen et al. 2021
*C. amphilecti *MC34	Glycerol	PHB	26/0.7	Shake flasks	Christensen et al. 2021
*C. amphilecti *MC34	Acetate	PHB	72/2.5	Shake flasks	Christensen et al. 2021
*C. amphilecti *MC34	Acetate+ valerate	P(3HB-co-14 mol% 3HV)	48/2.1	Shake flasks	Christensen et al. 2021
*Cobetia marina *DSM ­4741T	Glucose	PHB	46/1.1	Shake flasks	Christensen et al. 2021
*C. marina *DSM ­4741T	Glycerol	PHB	61/2.5	Shake flasks	Christensen et al. 2021
*C. marina *DSM ­4741T	Acetate	PHB	61/2.4	Shake flasks	Christensen et al. 2021
*C. marina *DSM ­4741T	Acetate+ valerate	P(3HB-co- 26 mol% 3HV)	59/3.7	Shake flasks	Christensen et al. 2021
*Billgrantia. campisalis*	Maltose	P(3HB-*co*−3.6 mol% 3HV)	45–81/ND	Shake flasks	Kulkarni et al. 2010
*Halomonas. campaniensis *LS21	Mixed substrate like kitchen waste	PHB	70/ND	Open fed-continuous	Yue et al. 2014
*H. campaniensis *LS21	Glucose	PHB	60/ND	7.5L Bioreactor	Jiang et al. 2017
*H. bluephagenesis* TD01	Glucose	PHB	89/ND	Open fed-batch and continuous	Tan et al. 2011
*Paracoccus sp. *LL1	Glucose	PHB	62/2.37	Shake flasks	Sawant et al. 2015

SDCLA seaweed-derived crude levulinic acid containing formic acid, residual sugars and dissolved minerals, ND not defined

## Discussion

The life of microorganisms under extreme conditions has led to the evolution of extremophiles with specific characteristics. This group of microorganisms includes halophiles inhabiting environments with high salt concentrations. They are found in saline lakes, salt marshes, saline soil, sea ice as well as salted foods such as fish, meat, and fermented products (Kanekar and Kanekar 2022). Many of these organisms show high biotechnological potential including biodegradable plastics production (Abdallah et al. 2025). The *Halomonadaceae* family is one of the most abundant groups of halophiles, and it was first introduced in the year 1988 by Franzmann et al. (1988) to accommodate moderately halophilic bacteria from the genera *Halomonas* and *Deleya*. Among the bacterial domain, members of the *Halomonadaceae* are the most used in the PHA production, such as *Vreelandella boliviensis*, *Halomonas bluephagenesis*, *Billgrantia campisalis*, *Vreelandella venusta*, *Halomonas halophila*, and many others (Angra et al. 2023).

The phylogenetic position of our isolate HP20-59 is not entirely clear; however, analyses based on the 16S rRNA gene sequence as well as the whole genome confirmed that it is a new species most closely related to the *Halovibrio* spp.

The genus *Halovibrio* was first proposed in 1988 and was isolated from the great Salt Lake, Utah, USA (Fendrich 1988). To date, several studies have addressed the classification of *Halovibrio* spp. (Dobson et al. 1993; Dobson and Franzmann 1996; Sorokin and Tindall 2006; de la Haba et al. 2023). Based on a phylogenetic study, de la Haba et al. (2023) demonstrated a significant evolutionary distance between *Halovibrio* spp. and other genera belonging to the *Halomonadaceae* family and suggested removing *Halovibrio* spp. from this family. They also suggested that the *Halovibrio* genus should be transferred to the family *Oleiphilaceae*. We also confirmed this result in our study. To date, no studies demonstrating PHA production in *Halovibrio* spp. have been published.

Halophilic bacteria offer several advantages such as reduced risk of microbial contamination due to their requirement for high salinity, the ability to utilize a wide range of low-cost substrates, and easier cell lysis during the PHA extraction process. (Yoo et al. 2024). Various rapid and inexpensive approaches have been used to screen PHA-producing microorganisms and visualize PHA granules over time (Kitamura and Doi 1994; Shenoy et al. 2012; Zribi-Maaloul et al. 2013; Koller 2015). Most of the published studies use transmission electron microscopy, which is an excellent tool for imaging PHA granules; however, it is less practical than cell staining due to its high cost, time-consuming, labour-intensive procedures, and need for highly qualified personnel (Canovas et al. 2021). Nile blue A was first used for PHA detection by Smith (1908) and Ostle and Holt (1982). The bacterial cell staining procedures include lipophilic dyes such as Nile Blue A, Sudan Black B, and Nile Red. Unlike Sudan Black B, Nile Blue A stain has higher sensitivity towards PHA-containing granules than other lipid-containing cell components and cell membranes (Ostle and Holt 1982; Wei et al. 2011; Mesquita et al. 2015). Later, many studies have reported the use of Nile Blue staining as a rapid and inexpensive method for PHA detection (Shenoy et al. 2012; Zribi-Maaloul et al. 2013; Koller 2015).

The qualitative spectrometric methods such as Raman, Infra-Red and nuclear magnetic resonance are the potential methods for structural characterization of PHAs (Koller and Rodríguez‐Contreras et al., 2015). Carbon source is known to have a crucial effect on the amount of PHAs produced in many species of the *Halomonadaceae* family (Lemos et al. 1998). Raman spectroscopy has advantages such as rapidity, non-invasive nature, minimal or no sample preparation, economically beneficial compared to chromatographic techniques, and can be used in microbial identification as well as in the field of chemistry, material sciences, mineralogy, art, and archaeology (Ciobota et al. 2010). Raman spectroscopy has been used in the identification and quantification of various PHA molecules (De Gelder et al. 2008; Samek et al. 2016; Jost et al. 2017). De Gelder et al. (2008) determined a representative Raman peak at approximately 1734 cm^−1^ exhibited by PHB isolated from *C. necator* DSM 428 and *C. necator* DSM 541.

Cost-effective industrial production of PHAs requires the use of affordable cultivation media, as some halophilic PHA-producing microorganisms depend on complex and expensive carbon and nitrogen sources (Koller 2015). The findings of this study indicate the potential of the HP20-59 isolate as an efficient and cost-effective PHA producer, especially in minimal media supplemented with low-cost carbon sources. Tomas et al. (2020) reported that *Halomonas* sp. SF2003 and *Cupriavidus necator* H16 exhibited varying levels of PHA production depending on the carbon source. PHA yields were 2.25 g L^−1^, 2.05 g L^−1^ and 1.02 g L^−1^, 2.25 g L^−1^ respectively using glucose and fructose. In contrast, the use of galactose resulted in a yield of 1.23 g L^−1^ of PHA in *Halomonas* sp. SF2003, whereas no detectable production was observed for *C. necator* H16. The *H. halophila* was reported with 4.58 g L^−1^, 4.85 g L^−1^, 4.98 g L^−1^, 3.41 g L^−1^, and 3.18 g L^−1^ PHA titre when cultivated in the presence of glucose, sucrose, rhamnose, galactose, and arabinose (Kucera et al. 2018). The *Vreelandella boliviensis* produces 0.55 g L^−1^ PHA in the presence of maltose and produces approximately 55% PHA in the presence of glucose and sucrose when cultivated in a 2-L fermentor (Quillaguaman et al. 2006). There are only a few studies investigating PHA production using ribose compared to glucose, sucrose, maltose, or xylose (Quillaguaman et al. 2006; Berlanga et al. 2012; Kucera et al. 2018). Stanley et al. (2018) reported the highest PHA yield in the presence of glucose (0.866 g L^−1^) in *Halomonas venusta* KT832796 cultivated on minimal medium, followed by fructose and sucrose (~0.7 g L^−1^). The use of glycerol resulted in a lower yield of PHAs (0.37 g L^−1^), while lactose did not support detectable PHA production. To conclude, our comparative analysis confirmed that the carbon source is the main factor determining the type and amount of PHA produced, which varies among bacterial species. In addition, class I PHA synthase was identified in analyzed genomes, and it seems the number of PHA-related gene copies did not affect PHA production (Table S2).

Our results indicate that the isolate HP20-59 is a promising candidate for PHA biosynthesis from various low-cost or waste-derived substrates. Although it lacks the ability to synthesize PHA directly from lactose, it demonstrates outstanding PHA production on glucose and galactose. Therefore, it can be effectively utilized for PHA production from lactose-rich feedstocks, such as cheese whey, following external enzymatic or chemical hydrolysis of lactose. Alternatively, in the near future, metabolic engineering strategies may enable the strain to cleave and utilize lactose directly (Guo et al. 2015). The HP20-59 strain, with its ability to grow in high salt concentrations and produce PHA from galactose, can be utilized to produce PHB using marine biomass rich in galactose, such as seaweed hydrolysates derived from *Eucheuma spinosum* and other red algae (Jung et al. 2022). Furthermore, the strain’s ability to metabolize sucrose supports its potential application in PHA production from sucrose-rich waste streams, such as sugarcane or sugar beet molasses (Naseem et al. 2024). Finally, the isolate’s utilization of glucose, galactose, xylose, and arabinose—major components of lignocellulose hydrolysates—further expands its potential for PHA production from renewable biomass sources.

The *phaC* gene cluster of the HP20-59 isolate shows horizontal gene transfer from the *Vreelendella* sp. and Zher Neoh et al. (2022) confirmed that *phaC* sequences and genome organization indicate active horizontal transfer of *phaC* within the *Halomonadaceae* family. PHA synthases of the *Halomonadaceae* family are very similar to class I PHA synthases, yet very different from well-studied PHA synthases of halophilic archaeons (Quillaguaman et al. 2010; Cai et al. 2011). According to Zher Neoh et al. (2022), class I *phaC* genes are also widely distributed in other families such as *Halomonadaceae* and class *Gammaproteobacteria*. Catone et al. (2014) reported that *Pseudomonas extremaustralis* contains a PHB gene cluster (phbF + PX) that exhibits a high similarity to genes found in the order *Burkholderiales*, suggesting its acquisition through horizontal gene transfer. A recent study by Kumar et al. (2018) suggests that partial *phaC* genes in some *Actinobacteria*, including genera *Rhodococcus*,* Arthrobacter*, and *Pseudarthrobacter*, exhibit a high degree of similarity to genes found in *Proteobacteria*. This finding indicates the occurrence of HGT at the intra-phylum level, highlighting potential evolutionary interactions between these bacterial groups.

A direct outcome of the current study is the discovery of a novel species *Halovibrio* sp. HP20-59 capable of PHA producing. According to the NCBI database, *Halovibrio* sp. HP20-59 is the second species in its genus, after *Halovibrio variabilis* NBRC 102410 (NZ_BJXV00000000), to possess the *phaC* gene. Since no studies have addressed PHA synthesis in *Halovibrio variabilis*, our study is the first published research on the genus *Halovibrio*. The obtained characteristics of the HP20-59 isolate raise interest in its classification in the future. The substrate preferences of the HP20-59 strain provide valuable insights to optimize culture conditions and media composition to enhance the efficiency in PHA production. Future research could focus on improving fermentation strategies and exploring alternative carbon sources to increase yield and cost-effectiveness.

## Conclusion

The *Halovibrio* strain HP20-59, isolated from Solivar near Prešov, Slovakia, has been identified as a novel species within the *Oceanospirillales* order, as confirmed by the 16S rRNA gene sequence and whole genome analysis. As a halophilic bacterium, the HP20-59 isolate shows significant potential for PHA production due to its ability to utilize diverse carbon sources, with galactose providing the highest PHA accumulation. In addition, the genome analysis indicates that the *phaC* gene in the HP20-59 strain likely originated from *Vreelandella* spp. via horizontal gene transfer, as evidenced by its conserved genetic organization. Generally, our study confirmed the key role of the type of carbon source in PHA production, rather than the structure of PHA-related genes. These findings improve our knowledge of halophilic PHA-producing bacteria and highlight the potential of *Halovibrio* sp. HP20-59 for sustainable industrial production of biopolymers.

## Supplementary Information

Below is the link to the electronic supplementary material.

ESM 1 (DOCX 1.09 MB)

## Data Availability

The data is available in the article and in the online supplementary material. The draft genome sequences of *Halovibrio* sp. HP20-59 are available in the GenBank database (https://www.ncbi.nlm.nih.gov/) under accession number NZ_JAWJUW000000000.2 (BioProject: PRJNA224116, BioSample: SAMN37745899) or as a WGS project JAWJUW02 (assembly GCF_033052795.2). The strain is available upon request for scientific purpose at the Department of Microbiology, Institute of Biology and Ecology, Faculty of Science, Pavol Jozef Safarik University of Kosice, Slovakia.
