# Supplementary material for: Genome sequence of Halovibrio sp. HP20-59 as a promising polyhydroxybutyrate producer
Source: Appl Microbiol Biotechnol. 2026 Jan 10;110:6. doi: 10.1007/s00253-025-13647-3 (PMC12791064; doi:10.1007/s00253-025-13647-3)
Supplement: Supplementary file 1 [file 253_2025_13647_MOESM1_ESM.pdf]

Genome sequence of *Halovibrio* sp. HP20-59 as a promising polyhydroxybutyrate producer

Shivani Adhvaryu[1], Jana Kiskova[1]*, Maria Piknova[1], Veronika Farkašová[1], Iva Buchtikova[2], Xenie Kourilova[2], Martin Kizovsky[3], Marketa Benesova[3], Ota Samek[3], Stanislav Obruca[2], Peter Pristas[1]

[1] Department of Microbiology, Institute of Biology and Ecology, Faculty of Science, Pavol Jozef Safarik University of Kosice, Srobarova 1014/2, 04180 Kosice, Slovakia.

[2] Institute of Food Science and Biotechnology, Faculty of Chemistry, Brno University of Technology, Purkynova 464/118, 61200, Brno, Czech Republic.

[3] Institute of Scientific Instruments of the Czech Academy of Sciences, Brno 61264, Czech Republic.

*Corresponding author. Department of Microbiology, Institute of Biology and Ecology, Faculty of Science, Pavol Jozef Safarik University of Kosice, Srobarova 1014/2, 04180 Kosice, Slovakia. E.mail: jana.kiskova@upjs.sk

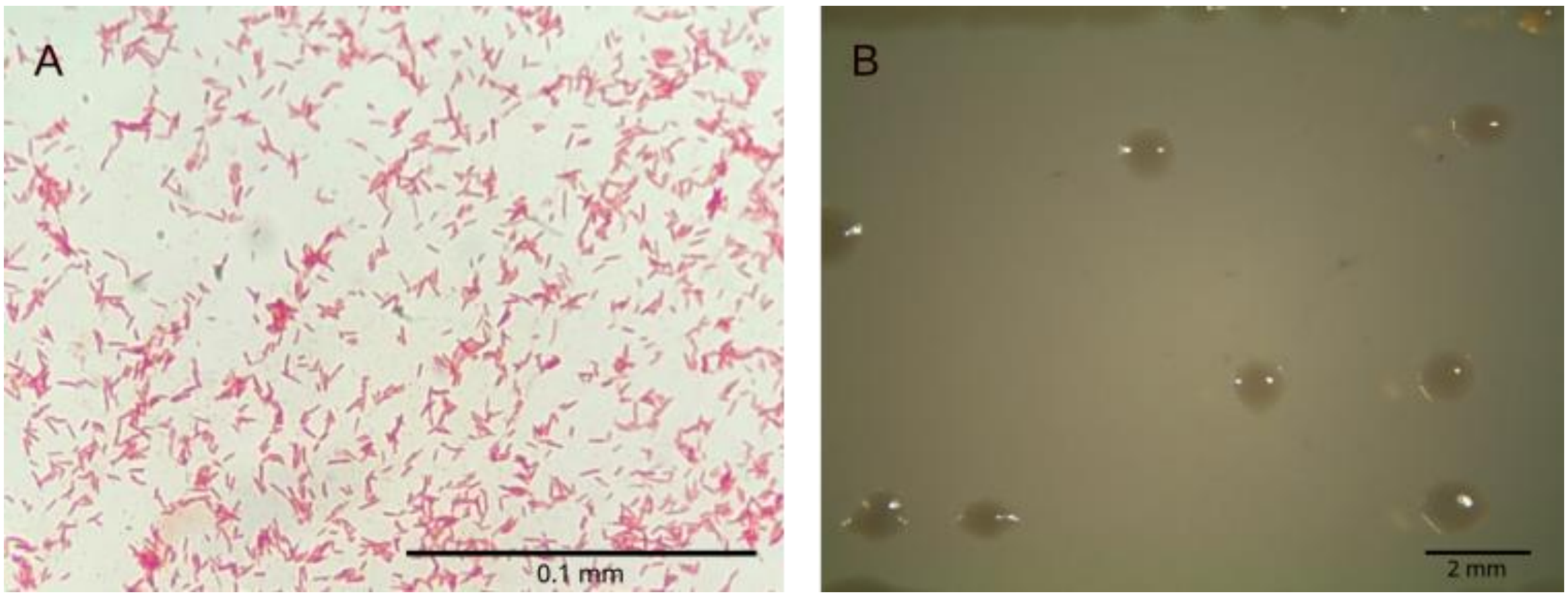

Fig. S1 Cell morphology (A) and colony characteristics (B) of the HP20-59 isolate obtained from the brine in Solivar (Prešov). Microscopic observation at 1000x (A) and 8x (B) total magnification

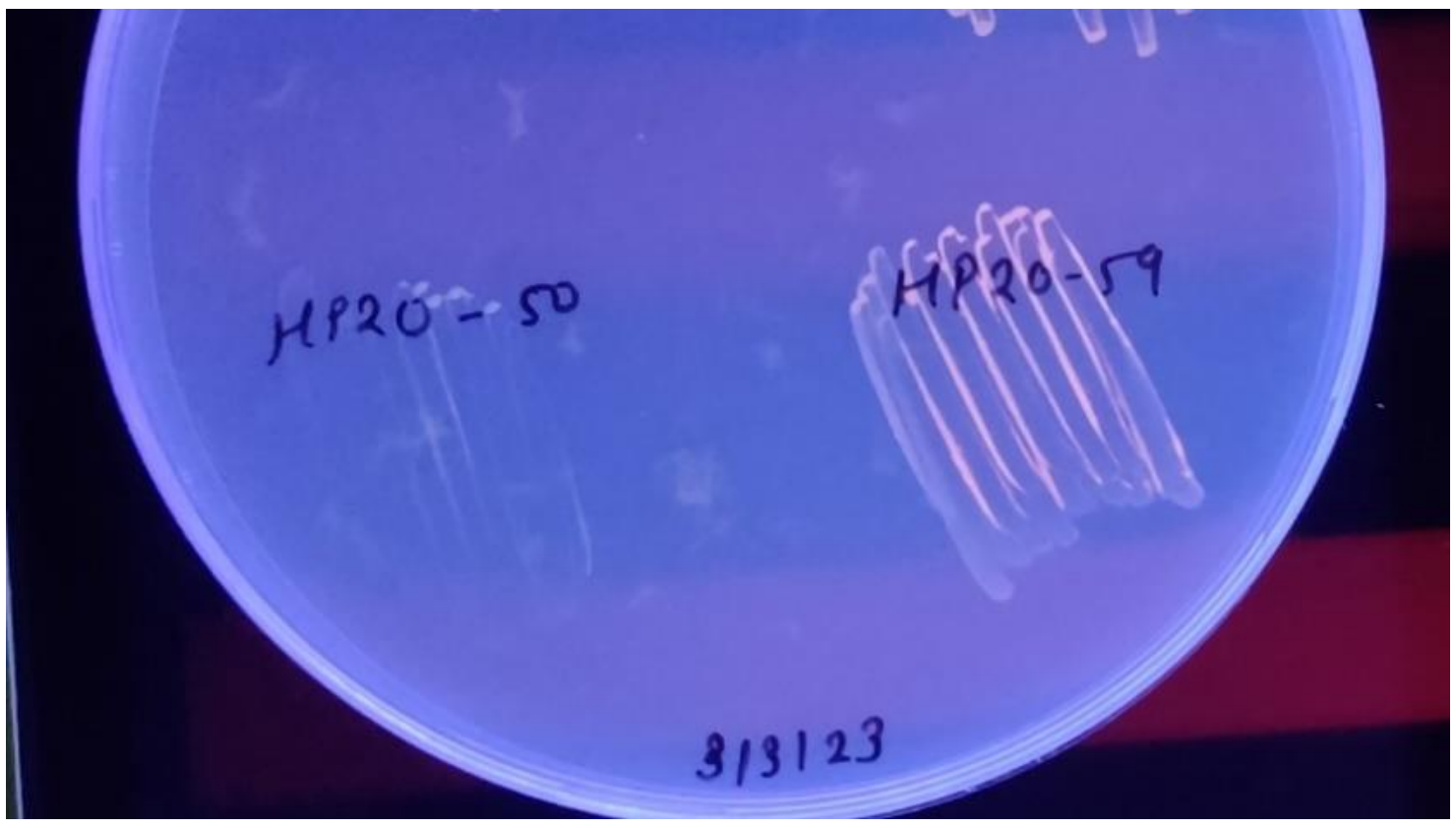

Fig. S2 The confirmation of PHA accumulation in the HP20-59 isolate using Nile Blue A under UV light

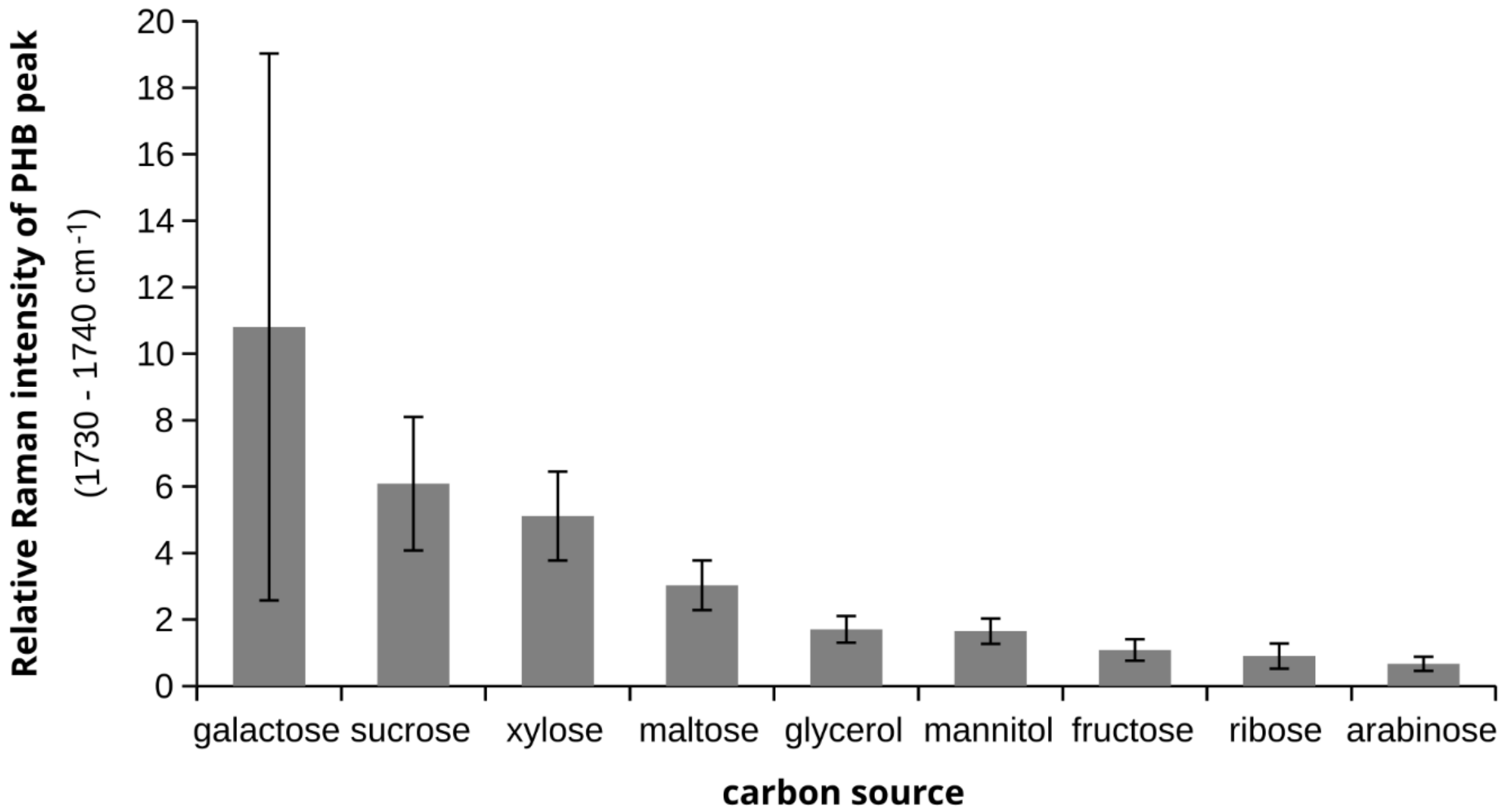

Fig. S3 Comparison of the PHB peak intensities (1730-1740 cm^{-1}) depending on the carbon source used in the cultivation medium

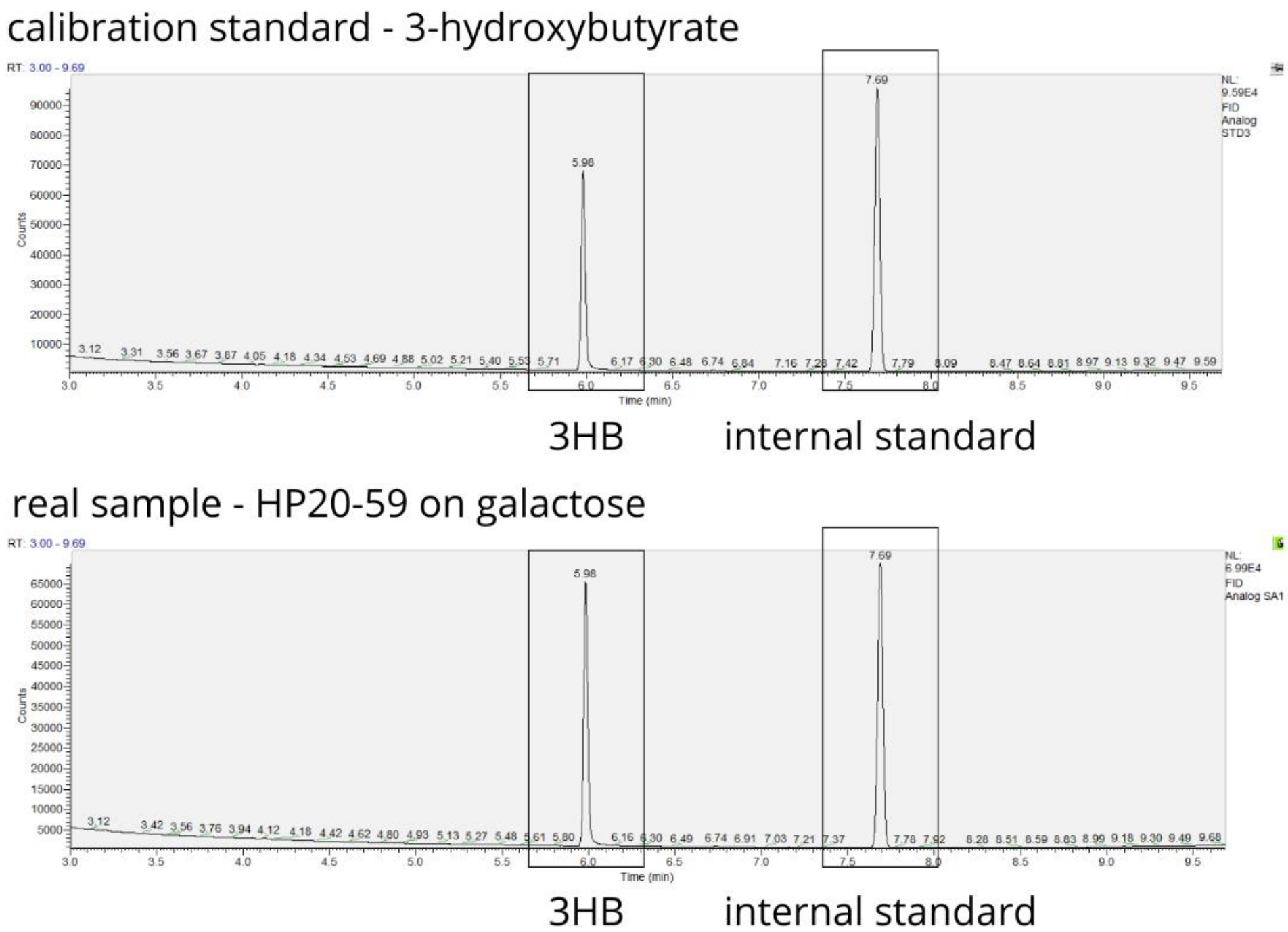

Fig S4 Representative chromatogram comparison assessed by GC- FID between standard 3-hydroxybutyrate (3HB) and the HP20-59 isolate in presence of galactose

Table S1 Genome features of the HP20-59 isolate according to the RAST server

**Features**	**HP20-59 isolate**
Domain	Bacteria
Taxonomy	Proteobacteria; Gammaproteobacteria; Oceanospirillales;
Genome size (bp)	4,165,293
Source	brine Solivar (Prešov, Slovakia)
GC content (%)	55.1
N50 (bp)	238926
L50	6
Number of contigs (with PEGs)	208
Number of subsystems	333
Number of coding sequences	4091
Number of RNAs	67

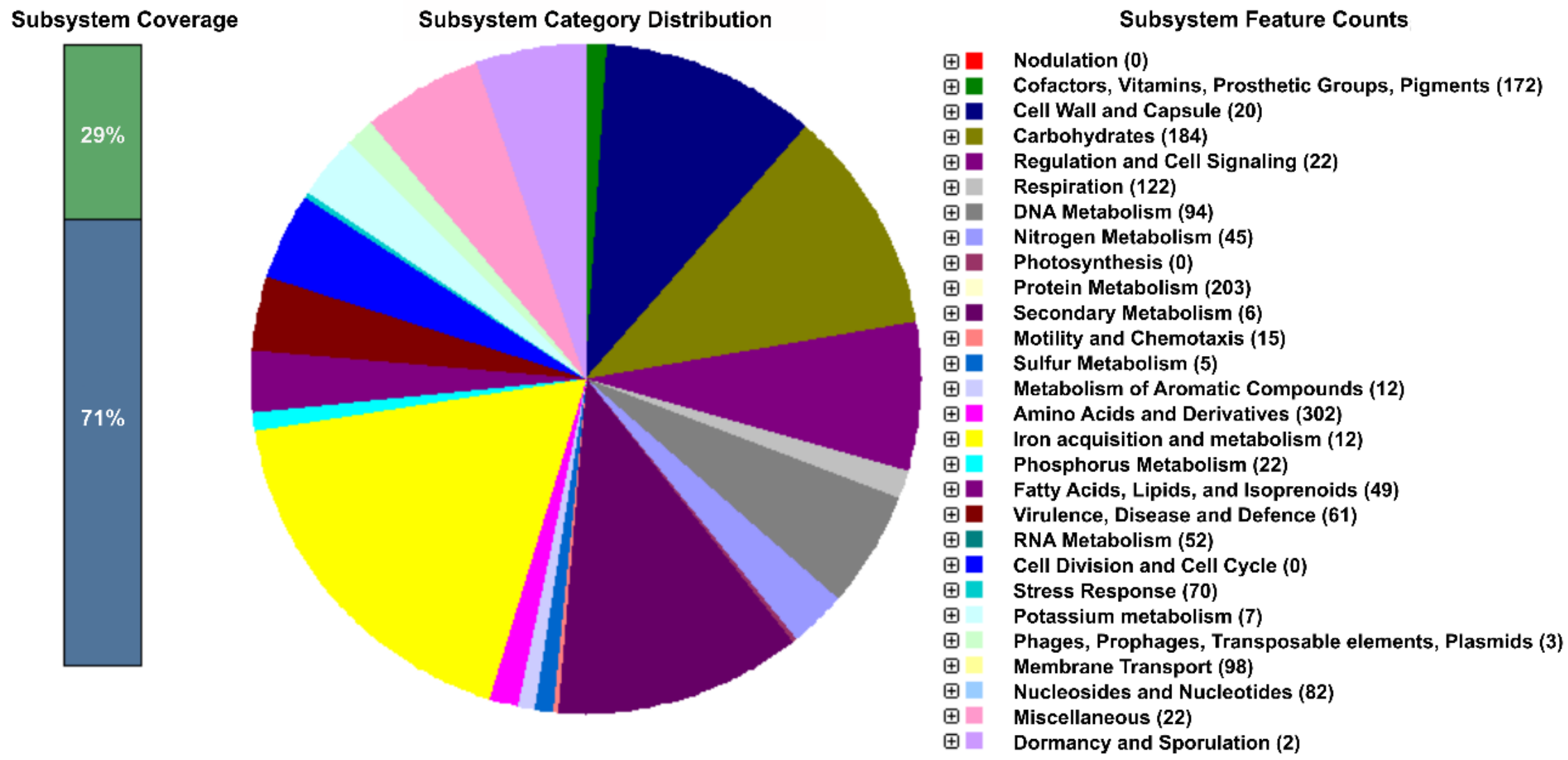

Fig. S5 Distribution of gene subsystems annotated in the HP20-59 genome.

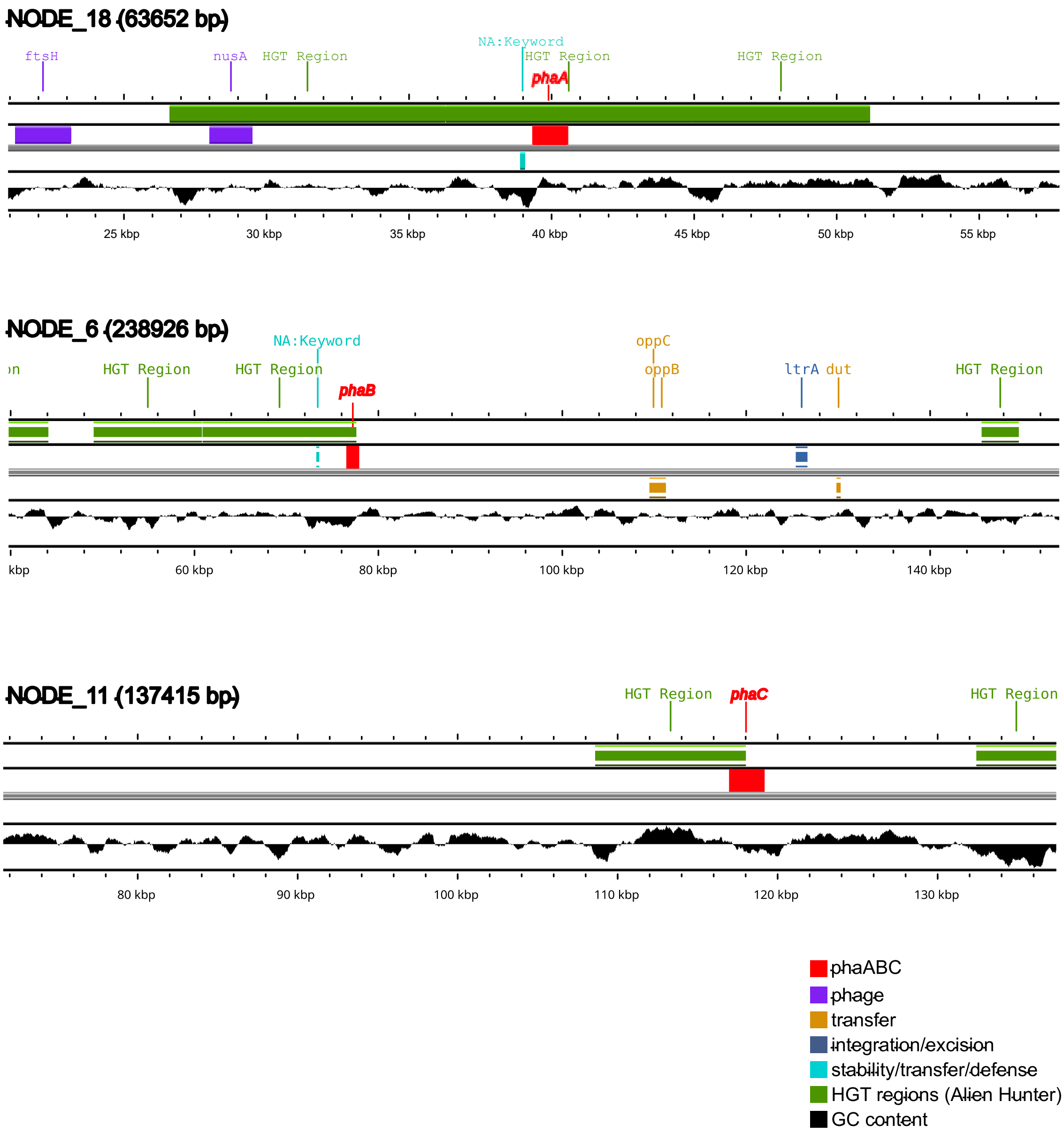

Fig. S6 PHA genes (red color) as a part of HGT regions located in the genome of *Halovibrio* sp. HP20-59

Table S2 Comparative genomic of PHA-related gene clusters in well-studied PHA producing bacteria

Bacterium	PHA production g L^{-1}	*phaA*	*phaB*	*phaC*[b]	*phaZ*	*fadA*	Reference
Halomonas sp. MC140	1.1	1[a]	1	1	1	1	Christensen et al. 2025
Halomonas halophila CCM 3662	4.59	1	1	1	-	1	Kourilová et al. 2021
Paraburkholderia sacchari DSM 17165	1.86	2	2	2	6	1	Kourilová et al. 2021
Caldimonas thermodepolymerans DSM 15344	4.26	1	1	1	1	3	Kourilová et al. 2021
Halomonas sp. YLGW01	1.6	1	1	1	1	1	Jeon et al. 2022

[a] The number of gene copies found in bacterial genome
[b] Class I poly(R)-hydroxyalkanoic acid synthase was found in all isolates studied

References

Christensen M, Chiciudean I, Lascu I, Jablonski P, Shapaval V, Zimmermann B, Tanase AM, Hansen H (2025) Halomonas sp. MC140, a polyhydroxyalkanoate (PHA) producer isolated from the Arctic environment. Sci Rep 15:23744. https://doi.org/10.1038/s41598-025-06898-7.

Kourilova X, Novackova I, Koller M, Obruca S (2021) Evaluation of mesophilic Burkholderia sacchari, thermophilic Schlegelella thermodepolymerans and halophilic Halomonas halophila for polyhydroxyalkanoates production on model media mimicking lignocellulose hydrolysates. Bioresour Technol 325:124704. https://doi.org/10.1016/j.biortech.2021.124704

Jeon JM, Son YS, Chang L, Yang YH, Yoon JJ (2022) Polyhydroxyalkanoate production by Halomonas sp. YLGW01 using volatile fatty acids: a statistical approach to apply for food-waste water. Biomass Conv Bioref 1:11. https://doi.org/10.1007/s13399-022-03550-6.
